# Morphological Convolutional Neural Network for Efficient Facial Expression Recognition

**DOI:** 10.3390/jimaging12040171

**Published:** 2026-04-15

**Authors:** Robert, Sarifuddin Madenda, Suryadi Harmanto, Michel Paindavoine, Dina Indarti

**Affiliations:** 1 Department of Informatic Engineering, Gunadarma University, Depok 16424, Indonesia; sarif@staff.gunadarma.ac.id (S.M.); suryadi_hs@staff.gunadarma.ac.id (S.H.); dina_indarti@staff.gunadarma.ac.id (D.I.); 2 LEAD, CNRS, UMR5022, Université Bourgogne Europe, 11 Esplanade Erasme, 21000 Dijon, France

**Keywords:** facial expression recognition, mathematical morphology, convolutional neural network, subject-independent evaluation, multi-source dataset, deep learning

## Abstract

This study proposes a morphological convolutional neural network (MCNN) architecture that integrates morphological operations with CNN layers for facial expression recognition (FER). Conventional CNN-based FER models primarily rely on appearance features and may be sensitive to illumination and demographic variations. This work investigates whether morphological structural representations provide complementary information to convolutional features. A multi-source and multi-ethnic FER dataset was constructed by combining CK+, JAFFE, KDEF, TFEID, and a newly collected Indonesian Facial Expression dataset, resulting in 3684 images from 326 subjects across seven expression classes. Subject-independent data splitting with 10-fold cross-validation was applied to ensure reliable evaluation. Experimental results show that the proposed MCNN1 model achieves an average accuracy of 88.16%, while the best MCNN2 variant achieves 88.7%, demonstrating competitive performance compared to MobileNetV2 (88.27%), VGG19 (87.58%), and the morphological baseline MNN (50.73%). The proposed model also demonstrates improved computational efficiency, achieving lower inference latency (21%) and reduced GPU memory usage (64%) compared to baseline models. These results indicate that integrating morphological representations into convolutional architectures provides a modest but consistent improvement in FER performance while enhancing generalization and efficiency under heterogeneous data conditions.

## 1. Introduction

Research on intelligent robotic systems has expanded rapidly in recent decades, driven by advances in industrial automation as well as emerging applications in healthcare, education, and social interaction [1,2,3,4]. While industrial robots such as welding and assembly systems are now widely deployed, increasing attention has been devoted to social robots designed to interact naturally with humans in everyday environments [4,5]. In such scenarios, non-verbal communication plays a crucial role, and facial expressions represent one of the dominant sources of information for conveying affective human–robot interaction [5]. Nevertheless, despite recent progress in dialogue systems and speech understanding, many robotic platforms remain limited in their ability to robustly interpret subtle facial cues and emotional dynamics in unconstrained real-world conditions.

Artificial intelligence (AI) has therefore been extensively adopted to address facial expression recognition (FER) and related visual perception problems [6,7]. Early FER pipelines were largely based on classical machine-learning approaches in which hand-crafted descriptors, such as Local Binary Patterns (LBPs), Gabor features, or Local Monotonic Patterns (LMPs), were combined with classifiers including Support Vector Machines (SVMs) or K-Nearest Neighbors (KNNs) [8,9,10,11,12]. Although such systems are computationally efficient and interpretable, their performance often degrades in the presence of noise, illumination changes, pose variation, or cross-dataset domain shifts. Deep learning (DL), particularly Convolutional Neural Networks (CNNs), has since transformed the field by enabling hierarchical feature learning directly from data, leading to substantial gains in recognition accuracy across many FER benchmarks [13,14,15,16].

In spite of these advances, CNN-based models frequently rely on large numbers of parameters and extensive training data, which can hinder deployment in real-time or resource-constrained robotic systems, particularly in terms of inference latency and memory usage. Moreover, convolutional filters predominantly capture appearance-dependent patterns such as illumination and skin tone, which may reduce robustness when applied to heterogeneous data. This trade-off between accuracy and computational efficiency has motivated increasing interest in hybrid approaches that embed prior knowledge about facial geometry or shape into learning pipelines. At the same time, recent advances in imaging research have increasingly emphasized mathematical morphology as an effective paradigm for capturing structural and shape-based characteristics in visual data. Classical grayscale morphology has been extended toward order-space formulations for multichannel imagery, illustrating the flexibility of morphological frameworks beyond single-channel representations [17]. Morphological operators have also proven valuable in biomedical imaging for quantifying tissue cellularity and structural organization in histological images [18].

Within facial analysis research, complementary directions have also emerged. Explicit modelling of facial muscle activity using the Facial Action Coding System (FACS) has inspired graph-based representations for real-time micro-expression recognition [19], while systematic investigations of expression categories have highlighted the importance of objective class definitions and annotation consistency for reliable benchmarking across datasets [20]. These studies underline two recurring themes in imaging-based affective computing: the enduring relevance of structural feature modelling and the central role of dataset design.

At the intersection of these trends, several recent works have proposed embedding morphological operators into deep neural networks. Morphology-aware convolutional models have demonstrated improved shape sensitivity in tasks such as hyperspectral image classification [21], whereas standalone Morphological Neural Networks (MNNs) have been shown to reduce parameter counts while maintaining competitive performance on datasets including MNIST and the German Traffic Sign Recognition Benchmark [22]. However, despite recent advances in deep CNN and transformer-based FER models that achieve high accuracy on benchmark datasets [13,16,23], many high-performing architectures, such as MobileNetV2 [24] and VGG-based models [16], primarily rely on appearance-driven feature learning, with limited emphasis on structural and geometric representations. As a result, it remains unclear whether morphology-based structural representations can provide complementary benefits beyond conventional convolutional features, particularly under heterogeneous data conditions. In this context, morphology-based operations are expected to provide complementary structural information that may enhance model robustness and computational efficiency.

Motivated by these open questions, this study proposes a novel FER framework that integrates morphological operators directly within a convolutional backbone, forming a Morphological Convolutional Neural Network (MCNN). In addition, a unified FER dataset is constructed by merging several widely used benchmarks, the Taiwanese Facial Expression Image Dataset (TFEID) [25], the Extended Cohn–Kanade dataset (CK+) [26,27], the Japanese Female Facial Expression dataset (JAFFE) [28], and the Karolinska Directed Emotional Faces dataset (KDEF) [29,30], together with a newly collected Indonesian Facial Expression (IFE) dataset. This fusion substantially increases both data diversity and demographic variability, enabling a systematic investigation of morphology-aware deep learning under varying conditions.

The main objective of this study is therefore twofold: (i) to evaluate whether morphological operations can enhance FER accuracy while improving computational efficiency relative to standard CNN baselines, and (ii) to analyze their behavior on a large, heterogeneous multi-dataset corpus. In addition to performance evaluation, this study examines whether morphological operations provide complementary structural information to convolutional features in facial expression recognition.

## 2. Methodology

### 2.1. Dataset Acquisition and Preprocessing

**Figure 1:**
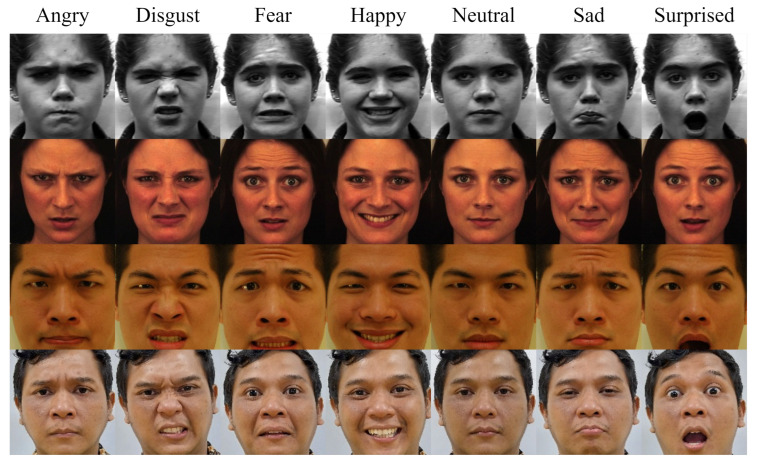
Sample facial expressions from the combined multi-source dataset.

In order to increase dataset diversity, this study constructs a multi-source dataset by combining publicly available datasets with a self-collected IFE dataset. The IFE dataset was collected from Indonesian participants under controlled acquisition conditions. Each subject was captured displaying seven basic facial expressions (angry, disgust, fear, happy, neutral, sad, and surprise) from multiple viewpoints to introduce intra-subject variability and improve robustness to pose variations. All data collection procedures followed ethical standards, and informed consent was obtained from all participants. The publicly available datasets include TFEID, JAFFE, CK+, and KDEF. The number of subjects in each dataset is as follows: CK+ (123), JAFFE (10), KDEF (140), TFEID (40), and IFE (13). Table 1 summarizes the distribution of images for each collected dataset, while Figure 1 shows sample facial expressions from the combined multi-source dataset.

During preprocessing, background noise such as text annotations in the CK+ dataset may affect model performance. Therefore, face detection using the Viola–Jones algorithm (VJA) was applied to ensure that each image contains only the facial region [31,32]. This step helps eliminate irrelevant information and standardizes the input for subsequent processing. Due to detection failures and false detections, 621 images (approximately 14.4% of the initial dataset) were excluded during this stage. These excluded samples correspond to cases where the face detector failed or incorrectly localized non-facial regions.

Since the proposed model employs morphological operations that require grayscale input, all detected facial images were converted from RGB to grayscale using the luminance (Y) component of the YC_b_C_r_ color space [33,34]. This conversion preserves structural information while reducing computational complexity. The detected face regions vary in size across datasets, whereas the model requires a fixed input size. Therefore, all images were resized to 160×160 pixels using bicubic interpolation while ensuring consistent input dimensions for the neural network [35]. After preprocessing, the total number of samples was reduced to 3684 images. Table 2 summarizes the distribution of images for each dataset and expression after preprocessing.

### 2.2. Splitting Dataset and Cross-Validation

To ensure a reliable and unbiased evaluation, a subject-independent data splitting strategy was adopted. In this approach, images belonging to the same subject were strictly separated across the training, validation, and testing subsets. This strategy is essential in FER tasks to prevent identity leakage, where the model may inadvertently learn subject-specific features due to the entanglement between identity and expression, resulting in overestimated performance and poor generalization to unseen individuals. Therefore, subject-independent evaluation is widely recommended as a standard protocol in FER research to ensure robust generalization across different subjects [11,36].

Model performance was evaluated using a 10-fold cross-validation scheme. The dataset was partitioned into ten subsets based on subject identity, ensuring that each fold contains mutually exclusive subjects. In each iteration, one subset was used for validation while the remaining subsets were used for training. The final performance metrics were computed as the average across all folds. This protocol provides a more reliable and statistically robust estimation of model performance compared to a single train–test split, particularly for heterogeneous datasets [37,38].

To ensure reproducibility, a fixed random seed of 42 was applied during data shuffling and partitioning. Reproducibility is an important aspect of deep learning research, as it enables consistent comparison and verification of experimental results across different runs [39].

No additional data balancing technique was applied in this study. As shown in Table 2, the class distribution after preprocessing does not exhibit significant imbalance. Applying aggressive balancing methods, such as undersampling, may reduce data diversity and potentially remove informative samples, particularly in multi-source FER datasets [40]. Therefore, the original data distribution was preserved to maintain representativeness.

Data augmentation was applied exclusively to the training set to improve generalization and reduce overfitting. The augmentation techniques included rescaling pixel values to the range [0, 1], horizontal flipping, zooming (up to 10%), width and height shifting (up to 5%), and brightness adjustment (80–120%). These transformations simulate realistic variations in facial appearance, such as pose, scale, and illumination changes, which are common challenges in FER tasks [40]. Rotation augmentation was not applied due to the limitations of the Viola–Jones face detection algorithm used in preprocessing, which is sensitive to rotational variations [31,32].

To further evaluate the generalization capability of the proposed model, a cross-dataset evaluation protocol was employed. In this setting, the model was trained on a combination of four datasets (CK+, JAFFE, KDEF, and IFE) and tested on a separate unseen dataset (TFEID). The testing dataset was kept completely independent and was not used during training or validation. This protocol simulates real-world conditions where training and testing data may come from different distributions and allows the assessment of model robustness under domain shift conditions, including variations in ethnicity, illumination, pose, and acquisition environments [41,42].

### 2.3. Mathematical Morphology

Mathematical morphology is a nonlinear image processing framework that analyzes geometric structures in images based on spatial relationships between pixels and structuring elements. Morphological operators emphasize structural patterns such as edges, contours, connectivity relationships, and shape characteristics within an image. The theoretical foundation of mathematical morphology was originally introduced by Serra and later extended to grayscale image analysis, forming the basis of many structural image processing techniques [43,44,45].

In this study, morphological operations are applied to grayscale facial images to emphasize structural characteristics of facial components that are relevant to facial expression recognition. Therefore, the morphological transformations are formulated within the grayscale mathematical morphology framework. Let $I_{f}:E\to R$ denote a grayscale image, where $E\subset Z^{2}$ represents the spatial domain of the image and $I_{f}\left(x\right)$ denotes the intensity value at pixel location x=(x,y). Morphological transformations are performed using a structuring element that defines a local neighborhood used to probe image structures [43,44].

The proposed method employs flat (binary) structuring elements, which specify only the spatial support of the neighborhood without assigning additional intensity weights to pixels. Therefore, although the input image is grayscale, the structuring elements are binary masks that determine the local neighborhood over which morphological transformations are applied. In this sense, the adopted formulation corresponds to grayscale mathematical morphology with flat (binary) structuring elements [45].

**Figure 2:**
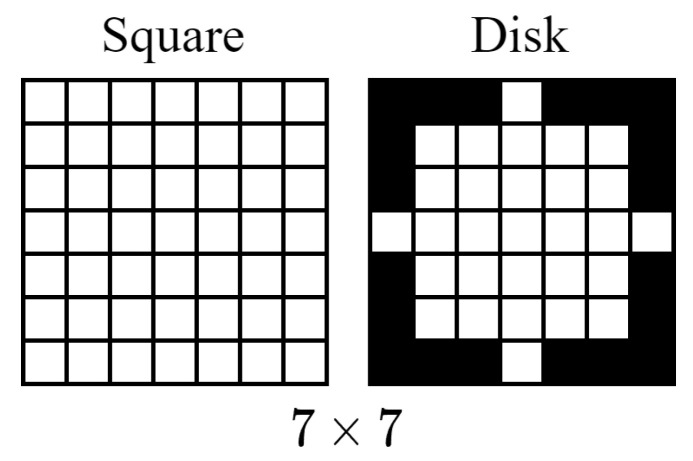
Example of 7×7 square-shaped and disk-shaped structuring element.

Two structuring element shapes are employed, namely square and disk, each with a size of 7×7 pixels [46]. The square structuring element captures horizontal and vertical spatial structures, while the disk-shaped structuring element preserves isotropic neighborhood structures. The size of 7×7 pixels was selected to capture meaningful local facial structures while avoiding excessive smoothing of fine facial details. For input images of size 160×160 pixels, a 7×7 structuring element covers a region large enough to capture the shape of eyebrows and mouth contours, yet sufficiently small to preserve subtle details such as eye corners or nasolabial folds, which are critical for discriminating between certain expressions (e.g., fear vs. surprise). Preliminary experiments with different structuring element sizes (3×3, 5×5, 7×7, 9×9, 15×15) indicated that the 7×7 configuration provided the best trade-off between structural sensitivity and feature preservation for facial expression patterns. Examples of the structuring elements used in this study are illustrated in Figure 2.

In grayscale mathematical morphology, the fundamental operators are dilation and erosion, which correspond to maximum and minimum transformations within the neighborhood defined by the structuring element [43,44,45,46]. Let $B\subset Z^{2}$ denote a flat (binary) structuring element and $B_{x}=\left\{x+y|y\in B\right\}$ represents its translation centered at pixel location *x*.

- Dilation expands bright structures and enhances object boundaries in an image. The grayscale dilation of image $I_{f}$ by structuring element *B* is defined as
  (1) $$I_{d}\left(x\right)=\left(I_{f}⊕B\right)\left(x\right)=\max_{y\in B_{x}}I_{f}\left(y\right)$$
  where ⊕ denotes the dilation operator. This operation assigns to each pixel the maximum intensity value within the translated neighborhood $B_{x}$ defined by the structuring element.
- Erosion performs the complementary operation of dilation by shrinking bright regions and suppressing small structures. The grayscale erosion is defined as
  (2) $$I_{e}\left(x\right)=\left(I_{f}⊖B\right)\left(x\right)=\min_{y\in B_{x}}I_{f}\left(y\right)$$
  where ⊖ denotes the erosion operator. This operation assigns to each pixel the minimum intensity value within the translated neighborhood $B_{x}$ defined by the structuring element.

More complex transformations can be constructed from dilation and erosion, which are opening and closing.

- Opening is defined as erosion followed by dilation:
  (3) $$I_{o}=I_{f}\circ B=\left(I_{f}⊖B\right)⊕B$$
  Opening smooths object contours, removes small bright structures, and eliminates thin protrusions while preserving the overall shape of larger regions.
- Closing is defined as dilation followed by erosion:
  (4) $$I_{c}=I_{f}\cdot B=\left(I_{f}⊕B\right)⊖B$$
  Closing fills small holes, connects nearby structures, and smooths object boundaries while preserving the geometric characteristics of larger regions.

To emphasize structural features relevant to facial expression recognition, morphological residual images are computed as the difference between the original image and morphologically transformed images. Since different morphological operators may increase or decrease pixel intensity values, directional subtraction is applied to avoid negative values and to preserve meaningful structural contrasts.

**Figure 3:**
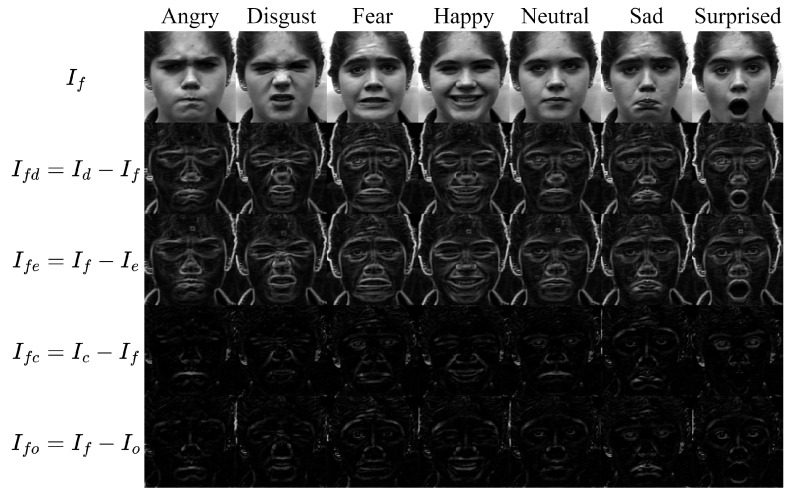
Example of morphological residual images, where each residual highlights different structural characteristics of facial expression components.

For dilation and closing operations, the transformed images generally produce higher intensity values than the original image. Therefore, the residual representations are computed as

(5) $$I_{fd}=I_{d}-I_{f}$$

(6) $$I_{fc}=I_{c}-I_{f}$$

where $I_{d}$ and $I_{c}$ denote the dilated and closed images respectively. For erosion and opening operations, the transformed images typically produce lower intensity values than the original image. Consequently, the residual representations are computed as

(7) $$I_{fe}=I_{f}-I_{e}$$

(8) $$I_{fo}=I_{f}-I_{o}$$

where $I_{e}$ and $I_{o}$ denote the eroded and opened images respectively. These residual images highlight geometric structures such as edges, contours, and deformation patterns of facial components. Since facial expressions are primarily characterized by geometric changes in facial regions such as the eyes, eyebrows, and mouth, emphasizing this structural information enables the model to focus on expression-related features while reducing sensitivity to illumination variations. Examples of morphological residual images generated using different morphological operators are shown in Figure 3, illustrating how dilation, erosion, opening, and closing emphasize complementary structural patterns in facial images.

As illustrated in Figure 3, different morphological operations emphasize distinct structural characteristics of facial components. A qualitative visual analysis of the residual images indicates that dilation and closing tend to highlight prominent facial regions such as the eyes, eyebrows, and mouth, whereas erosion suppresses homogeneous regions and preserves sharper structural boundaries. Opening generally produces weaker structural responses because small bright structures are removed during the erosion stage. These qualitative observations indicate that different morphological operators extract complementary structural features from facial images. Since facial expressions are primarily characterized by geometric deformations of facial components, emphasizing such structural patterns enables the model to focus on expression-related features while reducing sensitivity to illumination variations and skin tone differences.

### 2.4. Proposed MCNN and Baseline Models

#### 2.4.1. Proposed Morphological CNN (MCNN) Framework

This study proposes the MCNN framework that integrates mathematical morphology with convolutional neural networks to enhance structural feature representation for facial expression recognition. The main objective of this framework is to incorporate morphology-based transformations as a complementary feature extraction mechanism to conventional convolutional operations, as explored in morphology-aware deep learning approaches [21,22].

Unlike standard CNN pipelines that primarily rely on appearance-based features, the proposed framework explicitly emphasizes structural and geometric characteristics of facial components. This is achieved by generating morphological residual images derived from grayscale facial inputs using fundamental morphological operations, including dilation, erosion, opening, and closing, as described in Section 2.3. These residual representations highlight local structural variations such as edges, contours, and deformation patterns associated with facial expressions, encouraging the network to emphasize shape-related features rather than purely intensity-based patterns.

**Figure 4:**
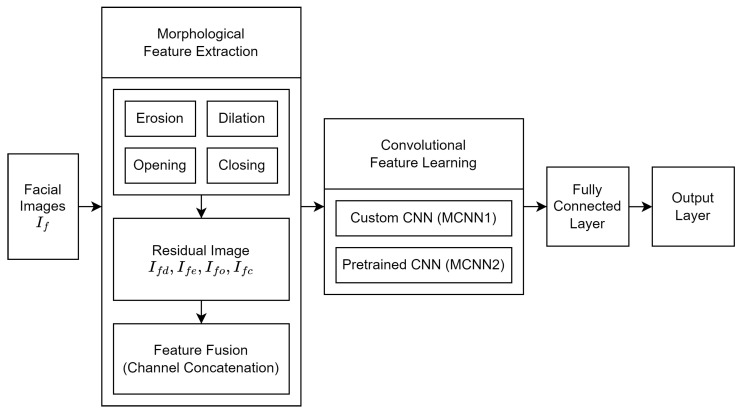
Overview of the proposed MCNN framework, illustrating the integration of morphological feature extraction, convolutional feature learning, and classification.

As illustrated in Figure 4, the MCNN framework consists of three main stages: (1) morphological feature extraction, (2) feature learning using convolutional layers, and (3) classification. In the first stage, the input image is transformed into a set of morphology-enhanced residual representations. Multiple residual images are generated and fused via channel concatenation as multi-channel inputs for the convolutional backbone. In the second stage, these representations are processed by a convolutional backbone to learn hierarchical features. Finally, the extracted features are mapped to expression classes using fully connected (FC) layers and a softmax classifier.

A key design principle of the proposed framework is flexibility with respect to the choice of convolutional backbone. The framework is designed to be compatible with both custom CNN architectures trained from scratch and pretrained deep networks. This enables a systematic investigation of morphology-based feature integration under different learning paradigms.

Based on this framework, two model families are developed in this study. The first, referred to as MCNN1, employs a custom convolutional architecture to evaluate the effectiveness of morphology-based representations in a fully controlled setting. The second, MCNN2, integrates morphological features into a pretrained MobileNetV2 architecture to examine their impact within a transfer learning framework. This separation allows controlled analysis of the contribution of morphology in both scratch-based and transfer learning settings.

#### 2.4.2. Proposed MCNN1 (Custom CNN Architecture)

To evaluate the effectiveness of morphology-based feature representations in a controlled setting, a custom convolutional neural network architecture, referred to as MCNN1, is developed and trained from scratch. Unlike pretrained models, MCNN1 enables direct assessment of the contribution of morphological operations without the influence of prior learned representations. This model is derived from the general MCNN framework described in Section 2.4.1, with a focus on a fully custom convolutional backbone.

**Figure 5:**
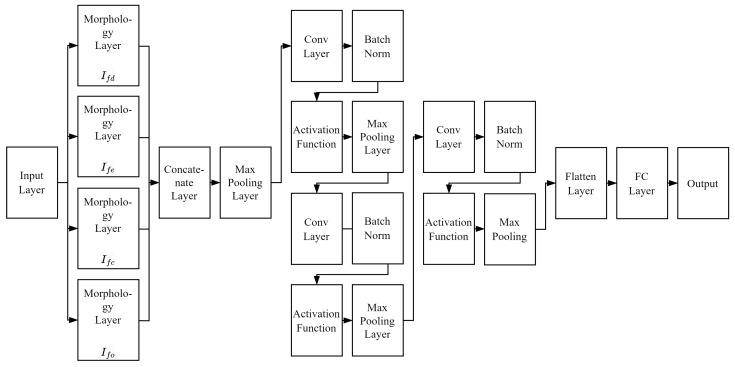
Illustration of the proposed MCNN1 architecture, showing the morphological feature extraction stage, channel concatenation, and the subsequent convolutional feature learning and classification layers.

As illustrated in Figure 5, the MCNN1 model takes a 160 × 160 grayscale facial image as input. The input is first processed through a morphological feature extraction stage, where four fundamental operations—dilation, erosion, opening, and closing—are applied, as defined in Section 2.3. Each operation produces a residual image through subtraction, namely dilation residual ($I_{fd}$), erosion residual ($I_{fe}$), opening residual ($I_{fo}$), and closing residual ($I_{fc}$). These residual representations emphasize structural variations such as edges, contours, and local deformations associated with facial expressions.

The resulting residual images are then combined through channel concatenation to form a multi-channel representation, which serves as the input to the convolutional network. The feature learning stage consists of four convolutional blocks, each comprising a convolutional layer followed by batch normalization, a Rectified Linear Unit (ReLU) activation function, and a max-pooling layer. The four convolutional blocks employ 128, 256, 512, and 1024 filters, respectively, enabling progressive extraction of hierarchical features from the morphology-enhanced inputs.

The output feature maps are subsequently flattened into a one-dimensional feature vector and passed through FC layers with ReLU activation. The final output layer consists of seven neurons corresponding to the seven facial expression classes, followed by a softmax activation function for classification. This custom architecture provides a lightweight and fully controllable framework for analyzing the contribution of morphology-based feature representations to FER.

#### 2.4.3. Proposed MCNN2

To further investigate the effectiveness of morphology-based feature integration within a transfer learning framework, a second model family, referred to as MCNN2, is developed by incorporating morphological representations into a pretrained MobileNetV2 architecture. Unlike MCNN1, which is trained from scratch, MCNN2 leverages pretrained weights initialized from ImageNet to capture high-level semantic features while integrating morphology-enhanced inputs.

MobileNetV2 was originally designed to accept three-channel RGB images [24]. However, in this study, facial images are represented in grayscale (single channel). To adapt the input to pretrained architecture while preserving morphological information, a multi-channel input strategy is employed. Specifically, the input to MCNN2 is constructed by combining the original grayscale image with selected morphological residual images, forming a three-channel composite input compatible with MobileNetV2.

**Figure 6:**
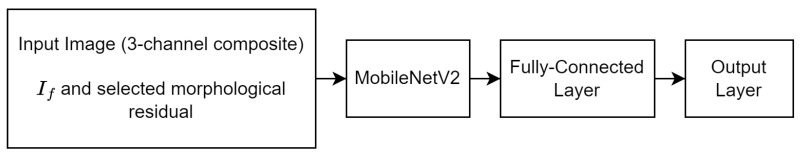
Unified architecture of the proposed MCNN2 models based on MobileNetV2.

As illustrated in Figure 6, the overall architecture of MCNN2 follows a unified design based on the standard MobileNetV2 backbone. In this framework, only the input representation is modified, while the backbone architecture remains unchanged. The morphological feature extraction process is identical to that described in Section 2.4.1, while the convolutional feature learning is performed using the pretrained MobileNetV2 network.

To systematically evaluate the contribution of different morphological operations, six input combinations are defined, as summarized in Table 3. Each combination corresponds to a distinct MCNN2 variant, constructed by pairing the original image with two morphological residual images. This design enables a controlled analysis of how different structural representations influence recognition performance.

Each input combination is fed into the MobileNetV2 backbone, which extracts hierarchical feature representations using depthwise separable convolutions. The resulting feature maps are then processed by FC layers followed by a softmax classifier to predict facial expression classes. This design enables a controlled comparison of different morphological feature combinations under a consistent backbone architecture, allowing the effect of each morphological operation to be systematically evaluated within a transfer learning setting.

#### 2.4.4. Baseline and Comparative Models (MobileNetV2, MNN, and VGG19)

To provide a comprehensive evaluation of the proposed MCNN framework, three baseline models are employed for comparison, namely MobileNetV2, VGG19, and a Morphological Neural Network (MNN). These models are selected to represent three distinct modeling paradigms: lightweight convolutional neural networks, deep high-capacity convolutional architectures, and morphology-based neural networks. This selection enables a systematic comparison between appearance-based, structure-based, and hybrid feature representations.

MobileNetV2 [24] is used as a lightweight CNN baseline due to its efficient architecture based on depthwise separable convolutions and inverted residual blocks. These design principles significantly reduce computational complexity while maintaining competitive performance. In this study, MobileNetV2 is initialized with pretrained weights and adapted to the FER task using the same input resolution (160 × 160) and training configuration as the proposed MCNN2 models. By keeping the backbone architecture unchanged and modifying only the input representation in MCNN2, this setup ensures that any observed performance differences can be attributed primarily to the inclusion of morphological features rather than differences in network capacity or training strategy.

VGG19 is included as a high-capacity deep CNN baseline, as commonly adopted in recent FER studies [16]. Compared to MobileNetV2, VGG19 employs a significantly deeper structure with a larger number of parameters, enabling the extraction of rich hierarchical features. The model is initialized with pre-trained ImageNet weights and fine-tuned on the target dataset. Following the transfer learning strategy described in [16], the early convolutional layers are frozen to preserve general low-level feature representations, while the deeper layers are trained to capture task-specific patterns related to facial expressions. This configuration provides a strong benchmark for evaluating whether morphology-enhanced representations can offer improvements over conventional deep CNNs.

In addition to CNN-based approaches, a MNN based on the work of Shen et al. [22] is employed as a structure-based baseline. Unlike convolutional neural networks, MNN utilizes morphological operations as the primary mechanism for feature extraction without relying on convolutional filters. Therefore, the model operates entirely in the morphological domain for feature extraction.

Consistent with the general formulation of MNN, the architecture consists of a morphological layer, a subtraction layer, a flatten layer, and fully connected layers. However, in this study, the architecture is adapted to align with the FER task and the preprocessing pipeline. Specifically, the model accepts 160×160 grayscale images as input and employs a fixed 7×7 disk-shaped structuring element in the morphological layer.

**Figure 7:**
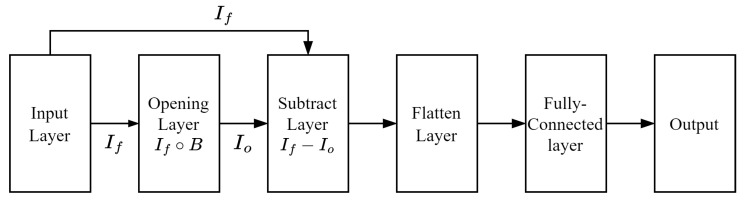
Adapted MNN architecture based on Shen et al. [22].

As illustrated in Figure 7, the implemented MNN uses an opening operation as the morphological transformation. The resulting image ($I_{o}$) is then combined with the original input image ($I_{f}$) through a subtraction operation ($I_{f}-I_{o}$), producing a morphological residual image ($I_{fo}$). This residual image emphasizes structural characteristics such as edges and geometric variations of facial components while suppressing homogeneous intensity regions.

Unlike the original MNN proposed by Shen et al. [22], which may incorporate multiple structuring elements and learnable parameters, the implementation in this study adopts a simplified configuration using a single fixed structuring element and a single morphological operation. This design choice ensures a controlled comparison with the proposed MCNN models, allowing the contribution of morphology-based representations to be more clearly isolated.

Since MNN relies solely on morphological transformations without convolutional feature extraction, it serves as a pure structure-based baseline. This enables a direct evaluation of whether integrating morphological operations into CNN architectures, as in the proposed MCNN framework, provides additional benefits over standalone morphology-based models.

To ensure a fair and consistent comparison, all baseline models are trained and evaluated under identical experimental conditions, including the same dataset, preprocessing pipeline, subject-independent data splitting, and evaluation metrics. Furthermore, the same optimization settings and training procedures are applied across models whenever applicable. This unified experimental setup ensures that performance differences can be attributed primarily to differences in feature representation and architectural design rather than variations in data or training configurations. Overall, the selected baseline models enable a comprehensive evaluation of whether morphological operations provide complementary structural information beyond conventional convolutional feature extraction.

### 2.5. Implementation Details and Training Configuration

All models were implemented using Python 3.10.11 with the TensorFlow/Keras framework and trained in a GPU-enabled environment equipped with an NVIDIA RTX 3060 Laptop GPU (6 GB memory) and 32 GB of RAM. To ensure reproducibility, a fixed random seed of 42 was applied consistently for dataset splitting, data shuffling, and data augmentation.

The input size for all models was set to 160×160 pixels, following the preprocessing procedure described in Section 2.1. For CNN-based models, input pixel values were rescaled from [0, 255] to [0, 1]. Morphological operations were applied to grayscale images as described in Section 2.3.

All models were trained using the Adam optimizer with an initial learning rate of 0.00001 and sparse categorical crossentropy as the loss function. A batch size of 32 was used, and training was conducted for a maximum of 100 epochs. Early stopping was applied based on validation performance, where the model with the best validation accuracy was selected.

For transfer learning models (MobileNetV2, VGG19, and MCNN2), pretrained weights from ImageNet were used for initialization. In MobileNetV2-based models, including MCNN2, the pretrained backbone was fine-tuned on the FER dataset. For VGG19, the transfer learning strategy followed [16], where early convolutional layers were frozen to preserve general low-level features, while deeper layers were fine-tuned to capture task-specific representations.

For MCNN2, the only modification compared to the baseline MobileNetV2 lies in the input representation. The original grayscale image was combined with selected morphological residual images to form a three-channel input. The backbone architecture and training procedure remained unchanged to ensure a fair comparison with the baseline MobileNetV2 model.

The MNN model was trained using the same optimizer, learning rate, batch size, and training schedule. Although MNN differs structurally from CNN-based models, identical training settings were maintained to ensure that performance differences are primarily attributed to architectural design rather than training configuration.

Model evaluation was conducted using a subject-independent 10-fold cross-validation, as described in Section 2.2. Performance metrics were computed as the average across all folds.

### 2.6. Evaluation Metric

To evaluate the performance of all models in this study, four evaluation metrics were employed: accuracy (training, validation, and testing), macro precision, macro recall, and macro F1-score. In addition, model performance is further analyzed using confusion matrix visualization to provide insights into class-wise predictions.

Accuracy (Acc) measures the overall proportion of correctly classified samples across all classes. As shown in Equation (9), it is computed by dividing the sum of true positives $\mathrm{TP}(C_{i})$ for each class $C_{i}$ by the total number of samples in the confusion matrix $C_{i,j}$.

(9) $$Acc\left(A_{\mathrm{reduced}}\right)=\frac{\sum_{i=1}^{N}\mathrm{TP}\left(C_{i}\right)}{\sum_{i=1}^{N}\sum_{j=1}^{N}C_{i,j}}$$

Precision (PPV) measures the ability of the model to avoid false positives. As shown in Equation (10), the macro-averaged precision is computed by taking the arithmetic mean of per-class precision $PPV(C_{i})$ over all *N* classes, giving equal weight to each class regardless of its sample size.

(10) $$PPV\left(macro\right)=\frac{1}{N}\sum_{i=1}^{N}PPV\left(C_{i}\right)$$

Recall (TPR) measures the ability of the model to detect all positive samples. As shown in Equation (11), the macro-averaged recall is computed by taking the arithmetic mean of per-class recall $TPR(C_{i})$ over all *N* classes, ensuring that minority classes contribute equally to the final score.

(11) $$TPR\left(macro\right)=\frac{1}{N}\sum_{i=1}^{N}TPR\left(C_{i}\right)$$

The $F_{1}$-Score is the harmonic mean of Precision and Recall, providing a balanced measure between the two. As shown in Equation (12), the macro-averaged $F_{1}$ score combines TPR(macro) and PPV(macro), penalizing models that sacrifice one metric for the other.

(12) $$F_{1}\left(macro\right)=2\cdot \frac{TPR(macro)\cdot PPV(macro)}{TPR(macro)+PPV(macro)}$$

In addition to classification performance, computational efficiency was also evaluated. Specifically, the total number of model parameters, inference latency, and peak GPU memory usage were measured. Inference latency was computed by averaging the prediction time over 100 repeated runs on a fixed test sample, while peak GPU memory usage was recorded during inference. These metrics provide additional insights into the practicality of the proposed models for real-world deployment.

## 3. Results and Analysis

### 3.1. Training Results

**Figure 8:**
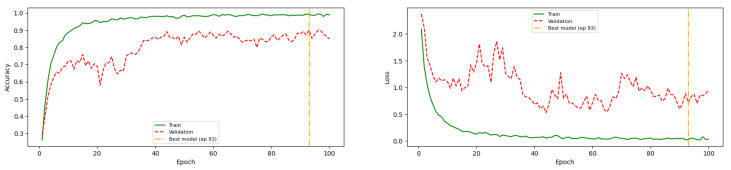
MCNN2v5 training history before tuning model, and only dropout applied.

During the training process, overfitting was identified as a primary challenge. As shown in Figure 8, the initial training configuration resulted in a noticeable gap between training and validation accuracy, indicating that the model tended to memorize training data rather than generalize effectively. Although dropout was applied as a regularization technique, it was insufficient to fully mitigate this issue.

To address overfitting, additional regularization strategies were introduced, including L2 regularization (1 × 10^−3^) and a learning rate reduction on plateau. As illustrated in Figure 8, these techniques helped reduce the gap between training and validation performance. However, they also led to a decrease in overall accuracy, suggesting a trade-off between generalization and model capacity.

**Figure 9:**
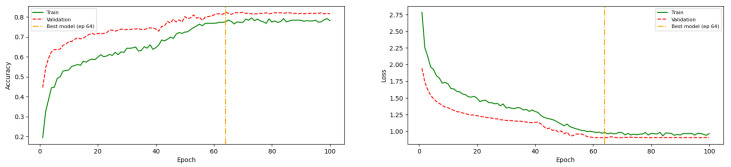
MCNN2v5 training history after L2 and learning rate reducer on plateau applied on the models.

Further tuning was therefore performed by reducing the complexity of the fully connected layers. Experimental results show that decreasing the number of neurons to 16 effectively improved generalization while maintaining competitive performance. As shown in Figure 9, the final configuration achieves a stable convergence between training and validation accuracy and loss, indicating that the model successfully balances fitting and generalization.

Given the significant influence of the learning rate on model performance, the learning rate reduction on plateau was subsequently removed. The tuning process then focused on the complexity of the dense layers by varying the number of neurons (ranging from 16 to 1024). Experimental results indicate that reducing the number of neurons to 16 in the dense layers effectively mitigates overfitting while preserving overall model performance.

**Figure 10:**
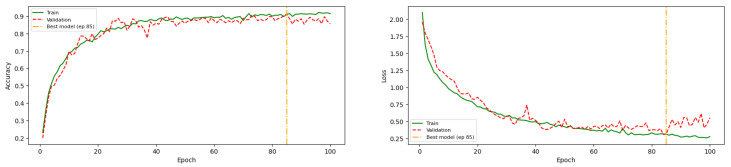
MCNN2v5 training history after tuning fully-connected layer complexity, demonstrates convergence between training and validation accuracy and loss.

Figure 10 presents an example of the training history after tuning. The results show that the training and validation accuracy and loss converge, and the selected model corresponds to the epoch indicated by the dashed orange vertical line.

Table 4 summarizes the average training accuracy, validation accuracy, training time, and number of parameters across all evaluated models using 10-fold cross-validation. Among the evaluated models, MCNN2 variants, particularly MCNN2v5, achieve the highest validation accuracy (90.82%), with performance comparable to MCNN2v4 and VGG19. Notably, the gap between training and validation accuracy for MCNN2 models is minimal, indicating strong generalization capability. In contrast, VGG19 exhibits a larger gap (greater than 6%), suggesting a higher tendency toward overfitting due to its high model capacity.

MCNN1 also shows a moderate gap (approximately 4%), indicating reasonable generalization performance but still less stable than MCNN2. On the other hand, the MNN model achieves significantly lower validation accuracy (51.92%) despite having a relatively large number of parameters. This suggests that the absence of hierarchical feature extraction mechanisms, such as convolution and pooling layers, limits its ability to capture discriminative facial features effectively.

In terms of model complexity, MobileNetV2 and all MCNN2 variants share the same number of parameters, as the morphological operations do not introduce additional learnable parameters. VGG19 has the largest number of parameters, while MNN also has a relatively high parameter count due to its architectural design. MCNN1 provides a balance between model complexity and performance.

Regarding training efficiency, MCNN2 variants demonstrate shorter training times compared to MobileNetV2, despite having the same parameter count. This indicates that morphology-enhanced inputs may simplify feature learning and improve optimization efficiency. MCNN1 also exhibits relatively fast training, likely due to its simpler architecture. In contrast, MNN shows the fastest training time, which can be attributed to its relatively shallow and linear structure.

Overall, these results indicate that integrating morphological representations into CNN architectures improves generalization while maintaining computational efficiency, particularly in comparison with high-capacity models such as VGG19.

### 3.2. Testing Results

In recent years, state-of-the-art FER methods based on deep convolutional neural networks and transformer architectures have demonstrated remarkable performance on benchmark datasets. For example, recent transformer-based approaches have reported very high recognition accuracy, reaching up to 99% on datasets such as CK+ under controlled experimental settings or dataset-specific conditions [23], which may not fully reflect cross-dataset or subject-independent scenarios. Existing approaches include both handcrafted and deep learning-based methods evaluated on benchmark datasets such as CK+ and JAFFE. For instance, LBP combined with Higher-Order Singular Value Decomposition (HOSVD) [11] focuses on person-independent facial expression recognition by integrating local and global texture features, achieving approximately 82% accuracy on the CK+ dataset under subject-independent evaluation. Meanwhile, more recent CNN-based approaches, such as the multi-layer feature recognition algorithm based on three-channel convolutional neural network (HFT-CNN) [13] and transfer learning-based models using architectures like VGG19 [16], are evaluated on datasets including CK+ and JAFFE, and report substantially higher accuracy, reaching up to 98% under certain experimental settings. However, these methods are typically evaluated under subject-dependent or random split protocols, where training and testing samples may share similar subject characteristics. As a result, the reported performance is strongly influenced by the evaluation protocol and may not fully reflect generalization capability in real-world scenarios. To address these limitations, this study adopts a more challenging and realistic evaluation setting, including subject-independent 10-fold cross-validation method which is then applied to the MobileNetV2, VGG19, and proposed MCNN1 and MCNN2 models across heterogeneous multi-source data.

Table 5 presents the testing performance of all evaluated models in terms of accuracy, precision, recall, and F1-score, averaged across 10-fold cross-validation. Overall, MobileNetV2 demonstrates balanced performance across all evaluation metrics, confirming its effectiveness as a lightweight baseline model under consistent experimental conditions. In contrast, the MNN model exhibits the lowest performance among all models, indicating that purely morphology-based architectures without convolutional feature extraction are insufficient for capturing complex facial expression patterns. VGG19 achieves competitive performance but remains slightly inferior to MobileNetV2, MCNN1, and all MCNN2 variants. The noticeable gap between training, validation, and testing performance further suggests a higher tendency toward overfitting, which is consistent with its large number of parameters and high model capacity. MCNN1 improves upon the MNN baseline, demonstrating the benefit of integrating morphological operations with convolutional layers. However, its performance remains slightly below that of MobileNetV2, indicating that models trained from scratch may be less effective than transfer learning-based approaches when handling heterogeneous multi-source datasets.

Among all evaluated models, the MCNN2 variants achieve the most consistent and competitive performance. In particular, MCNN2v5 achieves the highest testing accuracy (88.7%), with a slight improvement over MobileNetV2 (88.27%), indicating a modest but consistent performance gain. Several MCNN2 variants (v3, v4, and v5) slightly outperform MobileNetV2 while maintaining identical backbone architecture and training configuration. This suggests that the observed improvement is primarily attributed to the incorporation of morphological feature representations rather than differences in network capacity.

A more detailed analysis of the morphological components reveals that variants incorporating erosion (v1, v4, and v5) achieve slightly higher average accuracy compared to other combinations. This indicates that erosion plays an important role in enhancing discriminative features. Unlike dilation, which expands bright regions, erosion suppresses homogeneous areas while preserving structural boundaries such as eyelids, eyebrows, and mouth contours. Since facial expressions are largely characterized by geometric deformations rather than texture variations, this behavior enables the network to focus on structurally meaningful features.

In terms of computational efficiency, MCNN2 variants also demonstrate reduced training time compared to MobileNetV2, despite having identical parameter counts. This suggests that morphology-enhanced inputs may simplify feature learning and improve optimization efficiency. Additionally, the presence of zero-valued regions in morphological residual images may reduce computational overhead during convolution operations, contributing to faster convergence.

Although the improvement in accuracy is relatively small in magnitude, it is consistently observed across multiple MCNN2 variants. This indicates that morphology-guided representations provide complementary structural information that enhances model robustness without increasing model complexity.

**Figure 11:**
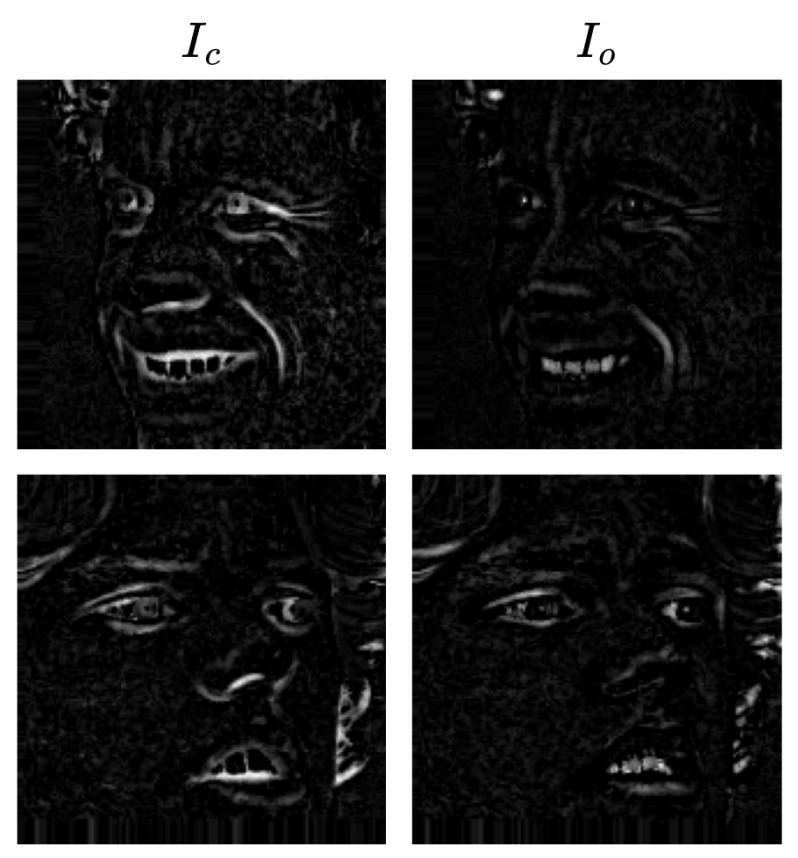
Feature maps visualization between closing and opening.

The comparison between MCNN2v4 and MCNN2v5 further highlights the importance of operator ordering. As illustrated in Figure 11, the feature maps generated by closing and opening exhibit distinct structural characteristics. Closing produces denser activations due to the initial dilation step, which may merge nearby structures and reduce feature discriminability. In contrast, opening first applies erosion, effectively suppressing noise and removing spurious activations before dilation restores the remaining structures. As a result, opening produces cleaner and more spatially localized feature maps that are better aligned with expression-related geometric patterns, leading to slightly improved recognition performance.

**Figure 12:**
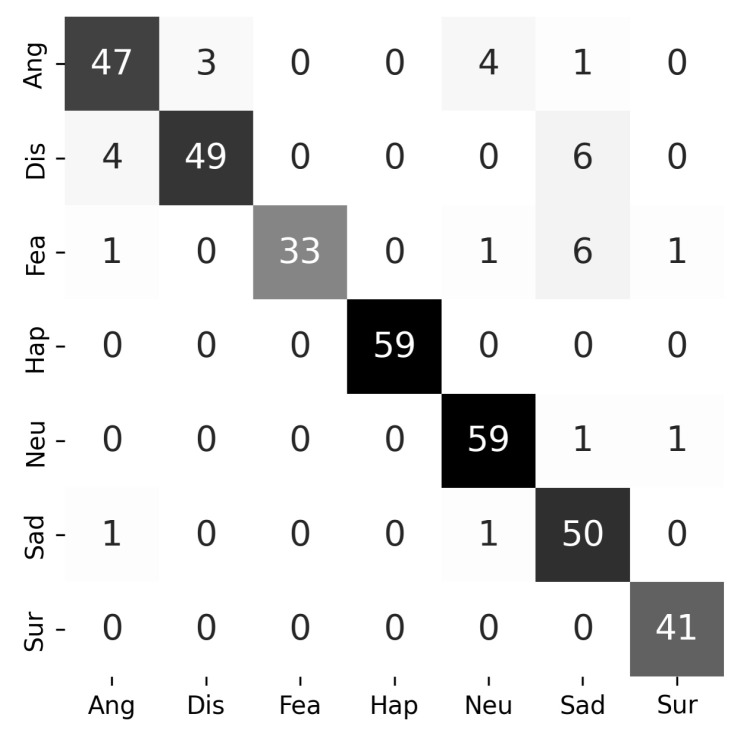
Confusion matrix of the proposed MCNN2v5 model under subject-independent 10-fold cross-validation. Darker colors indicate higher values.

To further analyze the class-wise performance of the proposed model, the confusion matrix of MCNN2v5 is presented in Figure 12. The results indicate that the model achieves high true positive rates across most expression classes, particularly for happy (59/59) and neutral (59/61), demonstrating strong discriminative capability for clearly distinguishable expressions. However, several misclassifications are observed in certain categories. In particular, fear is most frequently misclassified as sad (6 samples), while disgust is also confused with sad (6 samples). This suggests that the sad class acts as a confusion attractor, likely due to overlapping facial characteristics such as lowered eyebrows and subtle mouth deformations that are shared across multiple expressions. In addition, secondary confusion patterns are also observed. For example, disgust is misclassified as angry (4 samples) and angry as neutral (4 samples), indicating partial overlap in facial muscle activation patterns among these expressions. Although these errors occur less frequently than the dominant misclassifications, they highlight the remaining challenges in distinguishing expressions with similar structural features.

From a representation perspective, these findings are consistent with the behavior of morphological feature extraction. In particular, the use of erosion and opening operations in MCNN2v5 emphasizes fine structural details by suppressing noise and irrelevant intensity variations. This enables the model to better capture localized geometric patterns of facial components. However, expressions with highly similar structural configurations, such as fear and sad, may still produce overlapping feature representations, leading to residual confusion.

Overall, the misclassifications are limited and do not significantly affect overall performance. These findings indicate that the model effectively captures discriminative structural features while maintaining robustness across diverse expression categories.

### 3.3. Ablation Study

To evaluate the contribution of the proposed morphological feature extraction, ablation experiments were conducted on both MCNN1 and MCNN2 architectures. Results are reported as mean ± standard deviation across 10-fold cross-validation.

For MCNN1, the ablation study focuses on the role of the four morphological branches (dilation, erosion, opening, and closing) applied after the input layer. In the ablated configuration, these morphological operations are removed and replaced by simple channel replication, where the grayscale input image is duplicated four times before being passed to the convolutional layers. This design preserves the same input dimensionality and overall model complexity, ensuring a fair comparison where any observed differences can be attributed to the presence or absence of morphological transformations.

As shown in Table 6, the full MCNN1 model achieves a testing accuracy of 88% (±2%), while the ablated version achieves 87% (±4%). Although the improvement in mean accuracy is modest (approximately 1%), a more notable effect is observed in performance stability, where the standard deviation increases from ±2% to ±4% after removing the morphological components. This indicates that morphological residual representations contribute to more stable and consistent learning across folds.

In terms of computational cost, both configurations show similar performance. The inference latency of MCNN1 is 165 ms (±2), compared to 156 ms (±3) for the ablated version, while GPU memory usage and training time remain comparable. This suggests that the inclusion of morphological operations introduces only minimal computational overhead.

These results indicate that morphological residual representations provide complementary structural information that cannot be replicated by simple channel duplication. In particular, morphological operations emphasize geometric variations such as edges, contours, and local deformations of facial components, which are essential for distinguishing facial expressions.

For MCNN2, the ablation study aims to isolate the contribution of morphological operations within the transfer learning framework. The full MCNN2v5 model is compared against several variants: (1) removing erosion and replacing it with channel replication, (2) removing opening and replacing it with replication, and (3) removing both morphological operations entirely.

As presented in Table 7, the baseline MobileNetV2 achieves a testing accuracy of 88% (±3%), while MCNN2v5 achieves 89% (±2%), indicating a slight but consistent improvement. When either erosion or opening is removed individually, the performance remains approximately unchanged (89%), suggesting that each operation independently provides useful structural information. However, when both morphological operations are removed, the performance decreases to 88%, returning to the baseline level.

These findings suggest that the performance gain does not depend on a specific morphological operator, but rather on the presence of at least one meaningful morphological transformation that enriches the input representation with structural information.

In terms of computational efficiency, MCNN2v5 demonstrates substantial improvements compared to the baseline MobileNetV2. Specifically, inference latency is reduced from 281 ms to 222 ms (approximately 21% reduction), GPU memory usage decreases from 2231 MB to approximately 800 MB (approximately 64% reduction), and training time is reduced from 26 min to approximately 12 min (approximately 54% reduction). These results indicate that the proposed morphological pipeline provides a more efficient input representation without increasing model complexity.

From a representational perspective, the ablation results demonstrate that morphology-based transformations act as a structured preprocessing step that enriches grayscale inputs with meaningful geometric information. Unlike simple replication, which introduces redundant channels, morphological operations generate diverse feature maps that guide the network toward more discriminative representations.

Overall, the ablation study confirms that integrating morphological operations provides a modest but consistent improvement in performance, enhances model stability, and improves computational efficiency, while maintaining comparable model complexity.

### 3.4. Cross-Dataset Validation

To further evaluate the generalization capability of the proposed models, a cross-dataset validation experiment was conducted using a leave-one-dataset-out protocol. In this setting, the models were trained on a combination of CK+, JAFFE, KDEF, and IFE datasets, while the TFEID dataset was completely excluded from training and used exclusively for testing. This setup ensures that the testing data comes from a different distribution, allowing evaluation under domain shift conditions. Subject-independent splitting was applied to the training data to prevent any identity subject leakage between training and validation subsets. All experiments followed a 10-fold cross-validation scheme to ensure reliable performance estimation. Table 8 presents the cross-dataset validation results, including training, validation, and testing accuracy, as well as precision, recall, and F1-score.

As shown in Table 8, both MCNN2v5 and MCNN1 maintain strong performance when evaluated on the unseen TFEID dataset. MCNN2v5 achieves a testing accuracy of 89.28%, which is comparable to, and slightly higher than, its performance in the multi-source cross-validation setting. MCNN1 also demonstrates stable performance with a testing accuracy of 87.41%.

These results indicate that the proposed models generalize well across datasets with different characteristics, including variations in ethnicity, illumination, pose, and acquisition conditions. The consistent performance suggests that the learned representations are not overly dependent on dataset-specific features.

The strong cross-dataset performance of MCNN2v5 can be attributed to the incorporation of morphological feature representations. By emphasizing structural characteristics such as edges, contours, and geometric deformations of facial components, the model becomes less sensitive to appearance-based variations such as lighting conditions and skin tone differences. This allows the model to focus on expression-relevant features that are more invariant across datasets.

Furthermore, the use of subject-independent training and evaluation protocols ensures that the model learns expression-related patterns rather than subject-specific characteristics. This is particularly important in cross-dataset settings, where identity and data distribution may differ significantly between training and testing sets.

Although the observed improvement is modest in magnitude, the results are consistent with those obtained in the standard evaluation setting, indicating that morphology-guided representations contribute to robust and transferable feature learning.

Overall, these findings suggest that the proposed MCNN framework provides a reliable approach for facial expression recognition in real-world scenarios, where training and testing data may originate from different sources.

## 4. Conclusions

This study proposes a MCNN framework that integrates mathematical morphology with convolutional architectures for facial expression recognition. In addition, a multi-source and multi-ethnic dataset was constructed by combining several publicly available datasets with a newly collected IFE dataset, increasing both data diversity and evaluation robustness.

Experimental results demonstrate that the proposed MCNN2 models achieve competitive performance compared to conventional CNN baselines. In particular, MCNN2v5 achieves the highest testing accuracy (88.7%), with a slight but consistent improvement over MobileNetV2. While the performance gain is modest in magnitude, it is consistently observed across multiple configurations, indicating the effectiveness of morphology-guided feature representations.

The ablation study confirms that the integration of morphological operations contributes to improved stability and robustness, rather than merely increasing model complexity. The results show that morphology-based transformations provide complementary structural information that cannot be replicated through simple channel duplication.

Furthermore, cross-dataset validation demonstrates that the proposed approach generalizes well to unseen data, achieving 89.28% accuracy on a completely independent dataset. This suggests that morphology-guided representations help reduce sensitivity to appearance-based variations such as illumination and demographic differences, enabling more transferable feature learning.

In addition to performance improvements, the proposed MCNN framework also offers advantages in computational efficiency. The results show reduced inference latency, lower GPU memory usage, and shorter training time compared to baseline models, while maintaining comparable model complexity.

Overall, these findings indicate that integrating morphological representations into convolutional neural networks provides a modest but consistent improvement in facial expression recognition performance, while enhancing generalization and efficiency. The proposed approach offers a practical and effective solution for FER in heterogeneous and real-world scenarios.

Future work may explore more advanced strategies for integrating morphological operations into deep learning architectures, including adaptive or learnable structuring elements and sensitivity analysis of structuring element size. In addition, further evaluation will be conducted by incorporating additional benchmark datasets that are widely used in recent FER studies and performing direct comparisons under consistent evaluation protocols. This includes evaluating the proposed approach on commonly used datasets under subject-independent and cross-dataset settings to enable a more comprehensive and fair comparison with recent state-of-the-art methods. Furthermore, the proposed framework can be extended to other computer vision tasks involving structural feature analysis.

## Figures and Tables

**Table 1:** Distribution of collected images across facial expression classes and datasets.

Expression	CK+	JAFFE	KDEF	IFE	TFEID	TOTAL
Angry	45	30	420	65	34	**594**
Disgust	59	29	420	65	40	**613**
Fear	25	32	420	65	40	**582**
Happy	69	31	420	65	40	**625**
Neutral	122	30	420	65	39	**676**
Sad	28	31	419	65	39	**582**
Surprise	83	30	419	65	36	**633**
**Total**	**431**	**213**	**2938**	**455**	**268**	**4305**

Bold values indicate totals: row-wise for each facial expression, column-wise for each dataset, and the bottom-right value represents the overall total.

**Table 2:** Distribution of images after preprocessing across datasets and facial expression classes.

Expression	CK+	JAFFE	KDEF	IFE	TFEID	TOTAL
Angry	39	30	379	35	30	**513**
Disgust	58	19	408	55	36	**576**
Fear	25	23	348	24	17	437
Happy	69	30	410	60	25	**594**
Neutral	125	30	369	67	23	614
Sad	26	20	365	57	26	**494**
Surprise	82	29	256	63	26	**456**
**Total**	**424**	**181**	**2535**	**361**	**183**	**3684**

Bold values indicate totals: row-wise for each facial expression, column-wise for each dataset, and the bottom-right value represents the overall total.

**Table 3:** Input channel configuration of MCNN2 variants.

Model	Input Channels
MCNN2v1	$I_{f},I_{fd},I_{fe}$
MCNN2v2	$I_{f},I_{fd},I_{fc}$
MCNN2v3	$I_{f},I_{fd},I_{fo}$
MCNN2v4	$I_{f},I_{fe},I_{fc}$
MCNN2v5	$I_{f},I_{fe},I_{fo}$
MCNN2v6	$I_{f},I_{fc},I_{fo}$

**Table 4:** Model training summary.

Model	Train Accuracy	Validation Accuracy	Best Epoch at	Training Time	Total Parameter
MobileNetV2 [24]	89.28%	90.47%	75	26 m 3 s	2,283,783
MNN [22]	50.57%	51.92%	84	10 m 47 s	13,111,303
VGG19 [16]	96.64%	90.17%	63	24 m 21 s	23,439,687
MCNN1	94.12%	89.82%	83	11 m 8 s	6,426,951
MCNN2v1	89.07%	88.16%	78	11 m 21 s	2,283,783
MCNN2v2	88.90%	89.30%	84	12 m 8 s	2,283,783
MCNN2v3	88.64%	89.87%	79	11 m 54 s	2,283,783
MCNN2v4	89.44%	90.28%	76	12 m 16 s	2,283,783
MCNN2v5	89.51%	90.82%	74	12 m 12 s	2,283,783
MCNN2v6	88.65%	89.68%	78	12 m 37 s	2,283,783

**Table 5:** Model testing summary using different metric.

Model	Test Accuracy	Precision	Recall	F1-Score
MobileNetV2 [24]	88.27%	88.86%	87.62%	87.73%
MNN [22]	50.73%	49.39%	49.01%	47.87%
VGG19 [16]	87.69%	88.24%	87.25%	87.2%
MCNN1	88.16%	88.59%	87.60%	87.63%
MCNN2v1	87.89%	88.15%	87.33%	87.35%
MCNN2v2	87.81%	88.02%	87.34%	87.29%
MCNN2v3	88.30%	88.82%	87.73%	87.62%
MCNN2v4	88.46%	89.22%	87.80%	87.92%
MCNN2v5	88.70%	89.54%	88.62%	88.28%
MCNN2v6	87.92%	88.66%	87.32%	87.35%

**Table 6:** Ablation study of the proposed model MCNN1. Results are reported as mean ± standard deviation across 10 folds.

Configuration	Train (%)	Val (%)	Test (%)	Latency (ms)	GPU Usage (MB)	Train Time
MCNN1	94 ± 4	90 ± 2	88 ± 2	166 ± 2	824 ± 72	11 m 8 s ± 0 s
No morph + replicate	91 ± 5	90 ± 2	87 ± 4	156 ± 3	849 ± 156	11 m 22 s ± 0 s

**Table 7:** Ablation study of the proposed model MCNN2v5.

Configuration	Train (%)	Val (%)	Test (%)	Latency (ms)	GPU Usage (MB)	Train Time
MobileNetV2 (Baseline)	89±2	90±2	88±3	281±6	2231±23	26 m 3 s ± 31 s
MCNN2v5	90 ± 2	91 ± 2	89 ± 2	222 ± 4	800 ± 46	12 m 12 s ± 31 s
Remove erosion + replication	89 ± 2	91 ± 2	89 ± 3	224 ± 2	782 ± 92	12 m 12 s ± 41 s
Remove opening + replication	90 ± 2	91 ± 1	89 ± 2	221 ± 3	752 ± 110	12 m 14 s ± 32 s
Remove morph + replication	88±2	91±3	88±3	225±5	807±101	12 m 33 s ± 24 s

**Table 8:** Cross-dataset validation results (leave-one-dataset-out: trained on CK+, JAFFE, KDEF, IFE; tested on TFEID).

Model	Train (%)	Val (%)	Test (%)	Precision (%)	Recall (%)	F1-Score (%)
MCNN2v5	90.40	90.80	89.28	89.91	89.09	88.87
MCNN1	89.97	89.31	87.41	89.80	85.20	85.65

## Data Availability

The original contributions presented in this study are included in the article. Further inquiries can be directed to the corresponding authors.

## References

[1] Coman M.N., Rontescu C., Bogatu A.M., Cicic D.T. (2021). Analysis of robotic welding possibilities of a car chassis assembly. Iop Conf. Ser. Mater. Sci. Eng..

[2] Mukherjee D., Gupta K., Chang L.H., Najjaran H. (2022). A survey of robot learning strategies for human-robot collaboration in industrial settings. Rob. Comput.-Integr. Manuf..

[3] Casini S., Ducange P., Marcelloni F., Pollini L. (2025). Human-centered ai and autonomy in robotics: Insights from a bibliometric study. Proceedings of the 2025 International Joint Conference on Neural Networks (IJCNN).

[4] Lampropoulos G. (2025). Social Robots in Education: Current Trends and Future Perspectives. Information.

[5] Rawal N., Stock-Homburg R.M. (2022). Facial emotion expressions in human–robot interaction: A survey. Int. J. Soc. Robot..

[6] Janiesch C., Zschech P., Heinrich K. (2021). Machine learning and deep learning. Electron. Mark..

[7] Dalvi C., Rathod M., Patil S., Gite S., Kotecha K. (2021). A survey of ai-based facial emotion recognition: Features, ml & dl techniques, age-wise datasets and future directions. IEEE Access.

[8] Ullah S., Jan A., Khan G.M. (2021). Facial expression recognition using machine learning techniques. Proceedings of the 2021 International Conference on Engineering and Emerging Technologies (ICEET), Istanbul, Turkey.

[9] Dino H.I., Abdulrazzaq M.B. (2019). Facial expression classification based on svm, knn and mlp classifiers. Proceedings of the 2019 International Conference on Advanced Science and Engineering (ICOASE), Zakho-Duhok, Iraq.

[10] Ravi R., Yadhukrishna S., Prithviraj R. (2020). A face expression recognition using cnn & lbp. Proceedings of the 2020 Fourth International Conference on Computing Methodologies and Communication (ICCMC), Erode, India.

[11] He Y., Chen S. (2020). Person-independent facial expression recognition based on improved local binary pattern and higher-order singular value decomposition. IEEE Access.

[12] Chen B., Qu B., Zhou Y., Huang H., Guo J., Xian Y., Ma L., Yu J., Chen J. (2026). Llm-based pose normalization and multimodal fusion for facial expression recognition in extreme poses. J. Imaging.

[13] He Y., Zhang Y., Chen S., Hu Y. (2023). Facial expression recognition using hierarchical features with three-channel convolutional neural network. IEEE Access.

[14] Yang X., Fang Y., Raga Rodolfo C. (2024). Graph convolutional neural networks for micro-expression recognition—Fusion of facial action units for optical flow extraction. IEEE Access.

[15] Ren S., Sun M., Wang B., Liu M., Men S. (2025). High precision infant facial expression recognition by improved yolov8. IEEE Access.

[16] Kumar R., Corvisieri G., Fici T.F., Hussain S.I., Tegolo D., Valenti C. (2025). Transfer learning for facial expression recognition. Information.

[17] Sun S., Huang Y., Inoue K., Hara K. (2023). Order space-based morphology for color image processing. J. Imaging.

[18] Ortega-Ruiz M.A., Karabağ C., García Garduño V., Reyes-Aldasoro C.C. (2020). Morphological estimation of cellularity on neo-adjuvant treated breast cancer histological images. J. Imaging.

[19] Buhari A.M., Ooi C.P., Baskaran V.M., Phan R.C.W., Wong K., Tan W.H. (2020). Facs-based graph features for real-time micro-expression recognition. J. Imaging.

[20] Davison A.K., Merghani W., Yap M.H. (2018). Objective classes for micro-facial expression recognition. J. Imaging.

[21] Roy S., Mondal R., Paoletti M.E., Haut J.M., Plaza A. (2021). Morphological convolutional neural networks for hyperspectral image classification. IEEE J. Sel. Top. Appl. Earth Obs. Remote Sens..

[22] Shen Y., Shih F.Y., Zhong X., Chang I.C. (2022). Deep morphological neural networks. Int. J. Pattern Recognit. Artif. Intell..

[23] Nawaz U., Saeed Z., Atif K. (2025). A novel transformer-based approach for adult’s facial emotion recognition. IEEE Access.

[24] Sandler M., Howard A., Zhu M., Zhmoginov A., Chen L.C. (2019). MobileNetV2: Inverted residuals and linear bottlenecks. Proceedings of the IEEE Conference on Computer Vision and Pattern Recognition.

[25] Chen L.F., Yen Y.S. (2007). Taiwanese Facial Expression Image Database.

[26] Lucey P., Cohn J.F., Kanade T., Saragih J., Ambadar Z., Matthews I. (2010). The extended cohn-kanade dataset (ck+): A complete dataset for action unit and emotion-specified expression. Proceedings of the 2010 IEEE Computer Society Conference on Computer Vision and Pattern Recognition-Workshops.

[27] Kanade T., Cohn J.F., Tian Y. (2000). Comprehensive database for facial expression analysis. Proceedings of the fourth IEEE International Conference on Automatic Face and Gesture Recognition (Cat. No. PR00580).

[28] Lyons M., Akamatsu S., Kamachi M., Gyoba J. (1998). Coding facial expressions with gabor wavelets. Proceedings of the Third IEEE International Conference on Automatic Face and Gesture Recognition.

[29] Lundqvist D., Litton J. (1998). The Averaged Karolinska Directed Emotional Faces.

[30] Lundqvist D., Flykt A., Öhman A. (1998). Karolinska Directed Emotional Faces.

[31] Viola P., Jones M.J. (2001). Rapid object detection using a boosted cascade of simple features. Proceedings of the 2001 IEEE Computer Society Conference on Computer Vision and Pattern Recognition, CVPR 2001, Kauai, HI, USA.

[32] Viola P., Jones M.J. (2004). Robust real-time face detection. Int. J. Comput. Vis..

[33] Poynton C.A. (2003). Digital Video and Hdtv: Algorithms and Interfaces.

[34] Ahmad I., Moon I., Shin S.J. (2018). Color-to-grayscale algorithms effect on edge detection—A comparative study. Proceedings of the 2018 International Conference on Electronics, Information, and Communication (ICEIC), Honolulu, HI.

[35] Gonzalez R.C., Woods R.E., Masters B.R. (2009). Digital image processing, third edition. J. Biomed. Opt..

[36] Cîrneanu A.L., Popescu D., Iordache D. (2023). New trends in emotion recognition using image analysis by neural networks, a systematic review. Sensors.

[37] Kohavi R. (1995). A study of cross-validation and bootstrap for accuracy estimation and model selection. Proceedings of the International Joint Conference on Artificial Intelligence (IJCAI).

[38] Vabalas A., Gowen E., Poliakoff E., Casson A.J. (2019). Machine learning algorithm validation with a limited sample size. PLoS ONE.

[39] Pineau J., Vincent-Lamarre P., Sinha K., Lariviere V., Beygelzimer A., d’Alche Buc F., Fox E., Larochelle H. (2021). Improving reproducibility in machine learning research (a report from the neurips 2019 reproducibility program). J. Mach. Learn. Res..

[40] Shorten C., Khoshgoftaar T.M. (2019). A survey on image data augmentation for deep learning. J. Big Data.

[41] Li S., Deng W. (2022). Deep facial expression recognition: A survey. IEEE Trans. Affect. Comput..

[42] Zhang Z., Luo P., Loy C.C., Tang X. (2017). From facial expression recognition to interpersonal relation prediction. arXiv.

[43] Serra J. (1982). Image Analysis and Mathematical Morphology.

[44] Soille P. (2004). Morphological Image Analysis.

[45] Hermary R., Tochon G., Puybareau É., Kirszenberg A., Angulo J. (2022). Learning grayscale mathematical morphology with smooth morphological layers. J. Math. Imaging Vis..

[46] Robert, Madenda S., Harmanto S., Indarti D., Seyman M.N. (2025). Introduction to morphological neural network classification model for facial expression recognition. 3rd International Congress of Electrical and Computer Engineering.

